# Adaptive Enrichment of a Thermophilic Bacterial Isolate for Enhanced Enzymatic Activity

**DOI:** 10.3390/microorganisms8060871

**Published:** 2020-06-09

**Authors:** Tanvi Govil, Priya Saxena, Dipayan Samanta, Sindhu Suresh Singh, Sudhir Kumar, David R. Salem, Rajesh K. Sani

**Affiliations:** 1 Department of Chemical and Biological Engineering, South Dakota School of Mines and Technology, Rapid City, SD 57701, USA; Tanvi.govil@mines.sdsmt.edu (T.G.); dipayan.samanta@mines.sdsmt.edu (D.S.); 2 Composite and Nanocomposite Advanced Manufacturing—Biomaterials Center, Rapid City, SD 57701, USA; 3 Department of Biotechnology & Bioinformatics, Jaypee University of Information Technology, Solan, Himachal Pradesh 173215, India; Priya.saxena@mines.sdsmt.edu (P.S.); syalsudhir@gmail.com (S.K.); 4 Department of Nanoscience and Nanoengineering, South Dakota School of Mines and Technology, Rapid City, SD 57701, USA; sindhu.sureshsingh@mines.sdsmt.edu; 5 Department of Materials and Metallurgical Engineering, South Dakota School of Mines and Technology, Rapid City, SD 57701, USA; 6 BuG ReMeDEE consortium, Rapid City, SD 57701, USA

**Keywords:** adaptive laboratory evolution, laccase, lignocellulosic, *Geobacillus*, thermophile

## Abstract

The mimicking of evolution on a laboratory timescale to enhance biocatalyst specificity, substrate utilization activity, and/or product formation, is an effective and well-established approach that does not involve genetic engineering or regulatory details of the microorganism. The present work employed an evolutionary adaptive approach to improve the lignocellulose deconstruction capabilities of the strain by inducing the expression of laccase, a multicopper oxidase, in *Geobacillus* sp. strain WSUCF1. This bacterium is highly efficient in depolymerizing unprocessed lignocellulose, needing no preprocessing/pretreatment of the biomasses. However, it natively produces low levels of laccase. After 15 rounds of serially adapting this thermophilic strain in the presence of unprocessed corn stover as the selective pressure, we recorded a 20-fold increase in catalytic laccase activity, at 9.23 ± 0.6 U/mL, in an adapted yet stable strain of *Geobacillus* sp. WSUCF1, compared with the initial laccase production (0.46 ± 0.04 U/mL) obtained with the unadapted strain grown on unprocessed corn stover before optimization. Chemical composition analysis demonstrated that lignin removal by the adapted strain was 22 wt.% compared with 6 wt.% removal by the unadapted strain. These results signify a favorable prospect for fast, cost competitive bulk production of this thermostable enzyme. Also, this work has practical importance, as this fast adaptation of the *Geobacillus* sp. strain WSUCF1 suggests the possibility of growing industrial quantities of *Geobacillus* sp. strain WSUCF1 cells as biocatalysts on reasonably inexpensive carbon sources for commercial use. This work is the first application of the adaptive laboratory evolution approach for developing the desired phenotype of enhanced ligninolytic capability in any microbial strain.

## 1. Introduction

Second-generation lignocellulosic plant biomass (LCB) has a global yield of 150 billion tons per year [1], offering an abundant and steady source of renewable organic matter with important potential as an alternative to contemporary starch-based substrates in biorefineries [2]. However, their current usage in a biorefinery is hindered by the complex organic chemistry that exists between the cellulose, hemicellulose, and lignin components of the lignocellulose. Together, they form an intricate network, where lignin is particularly difficult to biodegrade and reduces the bioavailability of the other lignocellulose constituents. This poses a substantial challenge for the bioprocessing of lignocellulose, making it indispensable for the biomass to undergo a hydrolytic pretreatment before bioconversion [3]. Nonetheless, lignin can be depolymerized by “Laccases” (EC 1.10.3.2, benzenediol: oxygen oxidoreductase), an essential set of “eco-friendly” enzymes belonging to the multinuclear copper-containing oxidoreductases, produced widely in nature, by various higher plants and microorganisms [4].

To date, the most explored group of laccases is from fungi [5], which offers higher reduction of type I copper than bacterial laccases [6], as well as high yields. Nevertheless, the commercial exploitation of fungal laccases is usually hindered by longer fermentation period, excessive difficulty in controlling the glycosylation degree, and their applicability under a narrow temperature and pH range [4]. Based on many remarkable features compared to fungal laccases, there has been a swift upsurge in the usage and application of bacterial laccases in recent years. From an industrial perspective, the bacterial laccases (a) can function in a wide-ranging temperature and pH; (b) have a broad substrate specificity; (c) can be produced in a shorter time, leveraging the higher growth rates of the bacterial subsystems compared to their fungal counterparts; and (d) offer the flexibility for improvement of enzyme activity and expression level [7] owing to the ease of cloning and expression in the bacterial host through appropriate manipulation [4]. Indeed, bioprospecting of laccases from thermophiles has garnered particular attention for industrial processes, since bioprocessing of lignocelluloses by laccases at high temperature enables elevated rates of feedstock conversion, attributed to improved enzyme penetration and delignification attained at thermophilic reaction settings [8].

*Geobacillus* sp. strain WSUCF1, a thermophilic microbe obtained from the compost pile in Washington State University, Pullman, Washington, USA (46.7319° N, 117.1542° W), is one such bacterium that produces highly thermostable lignocellulose deconstruction enzymes when grown on unprocessed lignocellulosic substrates, such as corn stover and prairie cordgrass [9,10]. WSUCF1 strain is highly efficient in depolymerizing unprocessed lignocellulose, needing no preprocessing/pretreatment of the biomasses; hence, utilization of WSUCF1 as a whole cell, or even its cells extract (for enzyme cocktails), is an attractive target for thermophilic bioprocessing of lignocellulosic biomasses. In our earlier reports, we established that with their high thermostability, their activity over a wide range of temperatures, and their greater xylan hydrolysis than commercial enzymes, WSUCF1 crude xylanase (23.8 U/mL) [11] and crude xylosidase (147 U/mg) [12] are suitable for thermophilic lignocellulose bioconversion processes. What remains negative for WSUCF1, however, is the low expression for enzymes responsible for hydrolyzing cellulose (endoglucanase 0.022 U/mL, and β-glucosidase 0.51 U/mL) and lignin (laccase 0.096 U/mL) [13]. In our recent work, laccase from WSUCF1 was overexpressed in *Escherichia coli* using the pRham N-His SUMO expression system. The purified laccase had very high thermostability (t_1/2_ of 72 h at 70 °C) and low Km (0.146 mM)—the most thermostable reported so far—making the recombinant WSUCF1 laccase beneficial for numerous industrialized and biological applications that involve delignification [2]. However, the recombinant clone still had the production of the laccase enzyme at low concentrations (specific activity 1.73 U/mg: activity 0.037 U/mL) [2]. Indeed, low levels of laccase production by natural and genetically modified bacterial hosts, and the costs of producing bulk amounts constitute a bottleneck to industrial-scale applications of thermostable laccases [14]. In this work, to enhance the natural efficiency of *Geobacillus* sp. WSUCF1 to secrete laccase in higher amounts, we adopted a combined strategy of (1) optimizing high yielding and economical media for the production of laccase in WSUCF1 by testing different lignocellulosic substrates as the carbon source, as well as different lignin-based compounds as the mediators; and then (2) harnessing the mechanisms of evolution, adaptation, and selection, i.e., adaptive laboratory evolution (ALE), to improve the fitness of WSUCF1 strain via the application of a constant selection pressure, using the optimized media, constituents and culture conditions as the base.

In the US, about 1 billion dry tons of second-generation lignocellulosic material per year are available [15], of which corn stover (CS), with an average production of 550 million bushels annually, is the highest crop-generated lignocellulose, followed by soybeans, hay and wheat straw [16]. Moreover, in South Dakota, USA, the Black Hills region of the state is over 90% forested, with roughly 91% classified as “timber land”, and hence wood chips of ponderosa pine (the dominant species in the region) are available at an estimated 260,000 tons of residues per year [17]. Similarly, prairie cordgrass (PCG) is highly abundant in South Dakota and can be found in almost any wet or moist area [18]. In the present study, these lignocellulosic materials were therefore chosen to be tested as substrates for inducing laccase production in *Geobacillus* sp. strain WSUCF1.

ALE is a well-considered strain improvement strategy that is being used to deliver strains with novel phenotypes. It involves applying culture conditions that provide a selection pressure favoring the growth of mutants in the evolving population that confers the trait of interest [19]. In the past, this ability has been applied to improve yeast strains of two general categories: (1) evolutionary engineering of substrate utilization and product formation, and (2) evolutionary engineering of stress resistance. In the literature, there is only one example where this approach has been used towards enzyme formation, i.e., the evolutionary adaptation of an *Aspergillus niger* for enhanced cellulase production [20]. Hence, taking this classical approach as a base, in this study we developed a proof of concept ALE approach to evolve genetically modified yet stable, *Geobacillus* sp. strain WSUCF1 phenotypes with improved laccase, and lignocellulosic degradation capability. Attention was paid towards culturing a parental strain of *Geobacillus* sp. strain WSUCF1 in a detailed selective environment (a medium containing a high lignocellulose concentration as a selective pressure), directing the accretion of adaptation(s) towards anticipated lignocellulosic capability of this strain in the first place, via enhanced expression of laccase. To the best of our knowledge, this is the earliest effort where the adaptive evolutionary approach has been applied to generate an evolved bacterial strain of *Geobacillus* sp. strain WSUCF1 (eWSUCF1) with improved phenotypes towards laccase production.

## 2. Material and Methods

### 2.1. Strain, Culturing Conditions, and Sample Preparation

The *Geobacillus* sp. strain WSUCF1 was maintained at 4 °C and sub-cultured weekly on Luria broth agar plates at 60 °C prior to production experiments. For laccase production by *Geobacillus* sp. strain WSUCF1, a 2% (*v/v*) sample of the seed culture that had been grown at 150 rpm and 60°C for 48 h was inoculated into 500 mL of the Mineral Basal Salt Solution (MBSS) medium in a 1000-mL flask and incubated at 60°C and continuous agitation at 150 rpm for 10 days, with the content of the flasks harvested after every 24 h and assayed. Each liter of the seed medium contained 10 g tryptone, 10 g sodium chloride, and 5 g yeast extract. Each liter of the MBSS medium contained 0.25 g potassium nitrate, 0.1 g monopotassium phosphate, 0.1 g magnesium sulphate, 0.1 g yeast extract, 0.005 g sodium molybdate, and 0.02 g calcium chloride. In all the experimental setups, the initial pH of the medium was kept at 7.0, and to avoid the effect of adding different salts on the media pH, the culture medium pH was adjusted with 2 M solutions of HCl or NaOH as buffering agents.

### 2.2. Effect of Different Lignocellulose Materials on Laccase Induction in Geobacillus sp. Strain WSUCF1

Different natural substrates were screened with regard to their lignin contents to determine their appropriateness for improved laccase production using *Geobacillus* sp. strain WSUCF1. As a carbon source to be supplemented in the autoclaved MBSS media, the following lignocellulose substrates were tested: unprocessed prairie cord grass (PCG), pinewood (PW), and unprocessed corn stover (CS), each at a concentration of 1% (*w/v*). Later, with the lignocellulosic biomass giving the best laccase activity, experiments were repeated at varying concentrations of 0.25%,0.5%,1%, and 2% *w/v*. Prior to addition in the medium, the substrates were washed three to four times with distilled water to remove residual reducing sugars, boiled for 15 min, dried in an oven at 60 °C, and finally ground into fine powder. As a control, the media was supplemented with 2% glucose. The glucose stock solution of 20% in neutral distilled water was prepared and sterilized separately at 121 °C for 10 min, before being added into the media, which was autoclaved separately at 121 °C for 15 min.

### 2.3. Effect of Varying Co-Substrates and Inducers on Laccase Induction in Geobacillus sp. Strain WSUCF1

To assess the influence of distinct, refined co-substrates on laccase production, kraft lignin, beechwood xylan, and carboxy-methyl cellulose (CMC) at three different concentrations (0.025, 0.25, and 2.5% *v/v*) were incorporated into the MBSS medium. The control flask was bereft of any co-substrate. The fermentation was carried out for 10 days at 60 °C, initial medium pH 7.0, agitation speed 150 rpm, and CS as substrate at 0.5% *w/v*.

Similarly, to screen for supplementary inducers in the control medium, the following lignin monomers were tested: guaiacol, syringaldehyde, and 2,2′azino-bis (3-ethylbenzothiazoline-6-sulfonic acid) (ABTS), at five different concentrations (1 mM, 2 mM, 3 mM, 4 mM, and 5 mM). For this purpose, the stock solution of guaiacol, syringaldehyde, and ABTS were prepared in distilled water, and all the stock solutions were sterilized by filtration before use. The initial pH of every experimental set described above was adjusted to 7.0, and the temperature maintained at 60 °C, with agitation speed at 150 rpm. The control flask was void of any inducer but had the required concentration of CS as the substrate, and the appropriate co-substrate optimized in the previous experiments.

A further effect of metals ion concentration was studied by adding different concentrations of copper sulphate (1–5 mM), manganese chloride ion (1–5 mM), and hydrogen peroxide (0–2%) into the MBBS medium. The fermentation was carried out at 60 °C for 10 days, at pH 7.0, agitation speed at 150 rpm, with CS (0.5% *w/v*) as the substrate, and with the appropriate co-substrate and mediator optimized in the previous experiments. The control was devoid of any copper salt, manganese salt, or hydrogen peroxide.

### 2.4. Strain Adaptation

Adaptive evolution experiments were commenced from unadapted colonies of *Geobacillus* sp. strain WSUCF1 and were performed in triplicate in 250 mL Erlenmeyer flasks containing 100 mL of MBSS media with 0.5% (w/v) unprocessed corn stover as substrate and 0.025% kraft lignin as inducer at 150 rpm, pH 7, and 60 °C. Throughout the course of the adaptive growth, the concentration of inoculum at every single serial passage was adjusted to 5%, with each cycle continuing for 10 days, and from the last subculture, 100 ul of the sample was spread plated on a Luria Broth (LB) plate with 2% agar and incubated at 60 °C for 24 h. Next, from the last subculture, 10 single colonies were isolated, and the best evolved laccase producer among them (tested with laccase expression) was preserved at −80°C. Also, the best laccase producer *Geobacillus* sp. strain WSUCF1 mutant (designated eWSUCF1) was further inoculated into 100 mL of the enzyme induction media with 0.5% corn stover and 0.025% kraft lignin, and the expressed laccase enzyme wasquantified. The optimization experiments were performed in triplicate for each transfer, and data were averaged and presented as the mean ± standard deviation (SD).

### 2.5. Enzyme Production and Enzyme Activity

After incubation, the contents of the flasks were centrifuged at 10,000 rpm for 10 min, and the culture supernatants were used to assay the laccase enzyme activity. With respect to the experimental conditions where the pH of the medium was varied, care was taken to neutralize the pH of the supernatants before performing the enzyme assays. Laccase activity was determined by the oxidation of ABTS (2,2′-azino-bis (3-ethylbenzothiazoline-6-sulphonic acid)) (Sigma-Aldrich, St. Louis, MO, USA) buffered with 0.1 M sodium phosphate at pH 7.0. The reaction mixture (1 mL) contained equal volumes of enzyme extract and 1 mM ABTS prepared in 0.1 M sodium phosphate buffer (pH 7). The reaction mixture was incubated at 60 °C for 10 min, and absorbance was read at 420 nm in a spectrophotometer against a suitable blank. One IU (International Unit) of laccase activity was defined as the amount of the laccase that oxidized 1 μmol of ABTS substrate per min under given assay conditions. The enzyme activity was expressed in U/mL. The total protein content in the enzyme extracts was determined using Bradford reagent and BSA was used as standard [21]. Proper substrate and cell-free controls were prepared, and all the experiments were performed in triplicates, with data averaged and presented as the mean ± standard deviation (SD).

### 2.6. Biomass Composition Analysis

The amount (by weight) of cellulose, hemicellulose, and lignin content of the corn stover before and after its treatment with the parental *Geobacillus* sp. strain WSUCF1 and with the adapted WSUCF1 strain (designated eWSUCF1) was determined by following a National Renewable Energy Laboratory (NREL) laboratory analytical procedure [22], and the protocol developed by Goering and Van [23]. To observe the microstructural changes of the corn stover caused by the unadapted as well as the adapted strain of *Geobacillus* sp. strain WSUCF1, the corn stover samples were rinsed with water, then the dried samples were prepared by mounting on stubs for scanning electron microscopic analysis, using SEM, Zeiss Supra40, SDSMT, USA.

### 2.7. Solvents and Chemicals Used

All chemicals and media culture components were purchased from Sigma-Aldrich (St. Louis, MO, USA) and Fischer Scientific (Hampton, NH, USA). The solvents needed for performing biomass composition analysis were procured from ANKOM Technology (Macedon, NY, USA). Prairie cordgrass, pinewood, and corn stover was kindly provided by Dr. Zhisheng Cen from South Dakota State University, Brookings, SD, USA.

## 3. Results

### 3.1. Laccase Production from the Parental Strain

Adaptive laboratory evolution (ALE) has appeared as an influential tool in basic microbial research and strain development, where an organism is cultured in a condition of interest for many generations, and fitness (typically growth rate) is every so often upgraded as beneficial mutations are selected-for and accumulate. Process conditions and composition play essential roles in the successful laboratory adaptation of a bacterial strain like WSUCF1. Therefore, before conducting ALE experiments, it was first necessary to investigate the effects of the process parameters (e.g., temperature, pH, and time of harvesting) as well as different substrates, co-substrates, phenolic and non-phenolic inducers on laccase induction in *Geobacillus* sp. strain WSUCF1, and to subsequently use the optimized conditions to serially adapt the strain *Geobacillus* sp. WSUCF1 for higher production of laccase under thermophilic conditions. The results of the process parameter studies are provided in Section 3.2 and Section 3.3, and results from the ALE work are given in Section 3.5 and Section 3.6. Additionally, the results of the biomass degradation are given in Section 3.6.

The primary requirement for the evolution experiment was determined by assessing the growth of *Geobacillus* sp. strain WSUCF1 on various unprocessed lignocellulosic waste residues under varying concentrations and conditions, as the selection of the lignocellulosic material is a critical aspect for the cost-effective production of the ligninolytic enzymes.

Based on the regional availability of raw materials, three lignocellulosic biomasses (Corn Stover, CS; Prairie Cord Grass, PCG; and Pinewood, PW) were chosen to be tested as substrates for inducing laccase production in *Geobacillus* sp. strain WSUCF1. To start with, the experiments were carried out at 60 °C, and pH 7.0, which are the reported optimum fermentation temperature and pH for *Geobacillus* sp. strain WSUCF1 [8].

**Figure 1:**
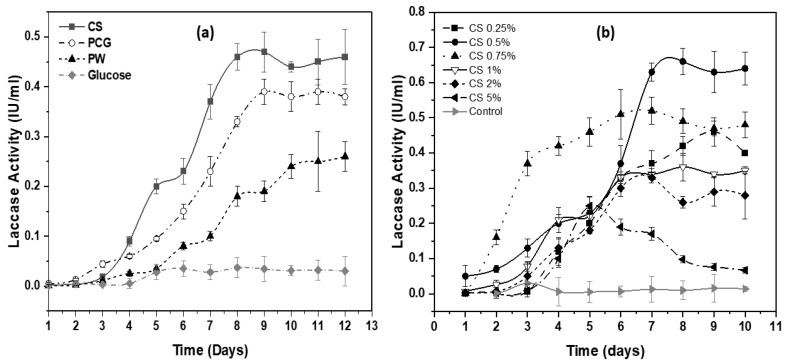
(**a**) Effect of agro-wastes as substrates on laccase production. (**b**) Effect of varying CS concentrations on laccase production in *Geobacillus* sp. strain WSUCF1 at 60 °C, pH 7.0, and agitation speed of 150 rpm, after 10 days of incubation. Here, CS= Corn stover; PCG = Prairie cordgrass; PW = Pinewood; IU = International Units. Note: All the experiments were performed in triplicates, with data averaged and presented as the mean ± standard deviation (SD).

At the tested concentration of 1% (w/v), all the screened lignocellulosic materials supported *Geobacillus* sp. strain WSUCF1 growth and produced laccase (Figure 1a). The maximum induction of laccase enzyme was attained for corn stover (CS) (0.46 ± 0.04 U/mL) on the 9th day of incubation. Prairie cordgrass (PCG) (0.39 ± 0.015 U/mL) on the 9th day of incubation also showed reasonable laccase activity, after following a more gradually increasing pattern. The least amount of enzyme production (0.26 ± 0.03 U/mL) was observed when pinewood, PW, was used in the medium. Generally, it is expected that the higher the lignin content in the substrate, the higher the amount of laccase production will be. However, our results had an inverse relationship between lignin and laccase induction, since PW has the highest amount of lignin, at 29%, compared to the amount of lignin in CS (16%), and PCG (21%) [24]. Since PW is expected to have high pentose content, PW hydrolysates produced during its degradation are rich in acetic acid and phenolic compounds and would be expected to cause cell growth inhibition from carboxylic acids [25]. The apparent discrepancy of low yields of laccase production and high lignin content for the PW substrate can therefore be attributed to strong inhibition to the growth of *Geobacillus* sp. strain WSUCF1 as a result of the hydrolysates produced during fermentation with PW. Since CS induced maximum laccase production, CS was chosen as the solid substrate for further experimentation.

When the parental strain of *Geobacillus* sp. strain WSUCF1 was inoculated into MBSS enzyme media with different concentrations of the unprocessed corn stover as a substrate (0.25%, 0.5%, 0.75%, 1%, and 2%), laccase production started after approximately 24 h in all cases, with maximum activity observed with 0.5% CS (0.66 ± 0.038 U/mL), followed by 0.75% CS (0.52 ± 0.038 U/mL), 0.25% CS (0.46 ± 0.04 U/mL), 1% CS (0.36 ± 0.04 U/mL) and, last, with 2% CS (0.33 ± 0.015 U/mL) (Figure 1b). In CS with 0.75% concentration, laccase activity appeared much faster than observed with any other concentration tested. However, activity was sustained at the highest IU only in the media with 0.5% *w/v* of CS. Laccase activities declined with time in media with 2% and higher CS concentrations. Notably, strongly diminishing enzyme activity was observed after 5 days in the media with 5% (*w/v*) CS, probably indicating the release of inhibitory substances such as weak acids, furaldehyde’s, and phenolics during the biomass degradation, negatively impacting the strain’s performance.

Since the highest laccase activity of 0.66 ± 0.038 U/mL was obtained with a CS concentration of 5 g/L, with both lower and higher CS concentrations showing poorer performance (Figure 1b), a medium with 0.5% *w/v* of unprocessed CS was selected for further studies, including the adaptation process. This CS preparation would be expected to impose considerable adaptation pressure on the cells without strongly inhibiting growth.

### 3.2. Effect of Addition of Co-Substrates and Mediators on Laccase Induction in Geobacillus sp. Strain WSUCF1

**Figure 2:**
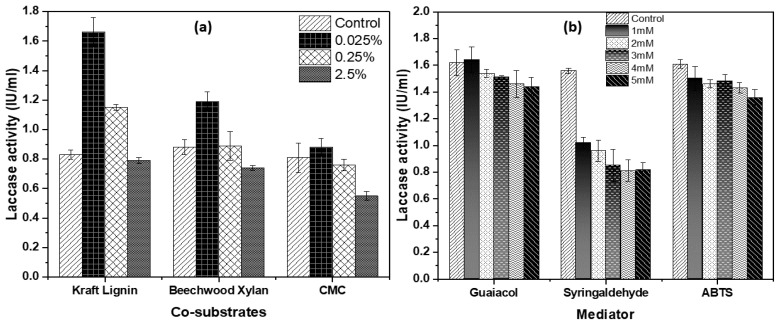
Effect of adding (**a**) co-substrates and (**b**) mediators on laccase induction in *Geobacillus* sp. strain WSUCF1 at 60 °C, pH 7.0, and agitation speed of 150 rpm, after 10 days of incubation with 0.5% (w/v) unprocessed corn stover (CS) as the carbon source. Here, CMC = Carboxymethyl cellulose, ABTS = 2,2′-azino-bis (3-ethylbenzothiazoline-6-sulfonic acid), IU = International Units. Note: All the experiments were performed in triplicates, with data averaged and presented as the mean ± standard deviation (SD).

The type and quantity of carbon sources in the culture medium are essential for the growth and production of enzymes by the bacteria. In this experiment, the effect of supplementing the CS media with different carbon co-substrates (kraft lignin, beechwood xylan, and CMC) on laccase production was evaluated, and the findings are shown in Figure 2a. The results showed that the addition of kraft lignin at 0.025% (*w/v*) enhanced the enzyme production almost twice from 0.83 ± 0.03 U/mL to 1.66 ± 0.1 U/mL, relative to the non-supplemented control medium (Figure 2a). The addition of beechwood xylan enhanced the enzyme production in *Geobacillus* sp. strain WSUCF1 by about 43% (at 0.025% beechwood xylan addition), compared with the non-supplemented control medium. However, when WSUCF1 was cultivated in the fermentation medium enriched with CMC, laccase expression did not significantly increase relative to the control. Thus, kraft lignin at 0.025% (*w/v*) was used as the co-substrate for the next round of enzyme production.

Next, the impact of mediators on laccase production was explored by supplementing the basal medium with guaiacol, syringaldehyde, and ABTS (Figure 2b). The results showed that compared to the control, all three mediators negatively affected the laccase activity, and these supplements were therefore dropped from inclusion in further experiments.

### 3.3. Effect of Metal Ion on Laccase Induction in Geobacillus sp. strain WSUCF1

**Figure 3:**
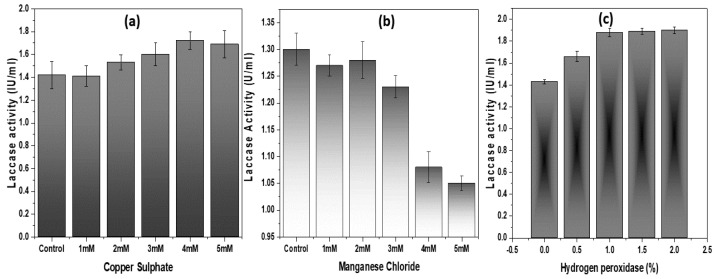
Effect of (**a**) Copper (Cu), (**b**) Manganese (Mn), and (**c**) hydrogen peroxidase on laccase induction in *Geobacillus* sp. strain WSUCF1 with 0.5% (w/v) unprocessed corn stover (CS) and 0.025% (w/v) kraft lignin, at 60 °C, pH 7.0, and agitation speed of 150 rpm, after 10 days of incubation. Here, IU = International Units. Note: All the experiments were performed in triplicates, with data averaged and presented as the mean ± standard deviation (SD).

To assess the impact of copper concentration on the activity of laccase, CuSO_4_ was added to the basal medium at varying concentrations. Since laccases are multicopper oxidases that catalyze the oxidation of lignin and its homologous compounds with the assistance of four copper atoms held within the catalytic core, it was expected that the use of Cu as a metal ion in the medium would boost induction of the laccase enzyme. However, as seen in Figure 3a, Cu has a very small effect on laccase production. The effect of Mn ions on laccase induction was also studied but, surprisingly, there was no obvious effect on laccase production (Figure 3b). Finally, the effect of adding hydrogen peroxide to the test media was investigated. In this case, the results indicate a small (but statistically significant) increase in enzyme activity in *Geobacillus* sp. strain WSUCF1 at hydrogen peroxide additions of 1% and higher (Figure 3c).

Overall, the results of these initial induction experiments suggest that CS may contain sufficient metal ions, phenolic, and non-phenolic constituents to induce the requisite laccases and/or peroxidases activity in *Geobacillus* sp. strain WSUCF1, and does not usefully benefit from extra phenolic compounds and/or metals as inducers in the media.

Through these initial optimization experiments, the parental thermophilic strain of *Geobacillus* sp. strain WSUCF1, a robust lignocellulose hydrolyzer, was made to express a higher level of laccase enzyme from unprocessed corn stover. Overall, after initial optimization of some of the cultural parameters such as pH, temperature, and the formulation of the culture medium, following one factor at a time (OFAT) methodology, the production of laccase enzyme was increased approximately four-fold from 0.46 ± 0.04 U/mL to 1.82 ± 0.098 U/mL. In the literature, researchers have taken this kind of optimization experiments further, making use of statistical tools, i.e., Plackett–Burman design and response surface methodology (RSM), which have proved advantageous in enhancing laccase production up to several-fold by analyzing the role of each factor critically [4]. The use of design of experiment to study the parameter interactions and evaluate additional parameters to further boost laccase production in *Geobacillus* sp. strain WSUCF1, is part of another manuscript (under preparation). In the present paper, we adopted ALE as an approach to adapt the *Geobacillus* sp. strain WSUCF1 to improve its native delignification and hydrolysis capability.

### 3.4. Laboratory Adaptive Evolution (ALE)

**Figure 4:**
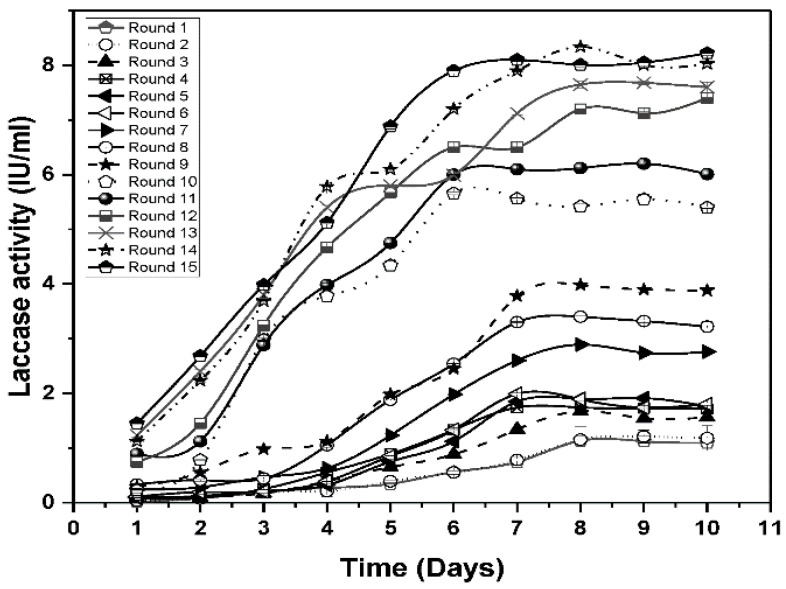
Maximal laccase activity in adapted strains of *Geobacillus* sp. strain WSUCF1 (eWSUCF1) during laboratory adaptive evolution with 0.5% (*w/v*) unprocessed corn stover (CS) and 0.025% (*w/v*) kraft lignin, at 60 °C, pH 7.0, and agitation speed of 150 rpm, after 10 days of incubation. Here, IU = International Units. Note: All the experiments were performed in triplicates, with data averaged and presented as the mean ± standard deviation (SD).

To further improve the performance of *Geobacillus* sp. strain WSUCF1, we serially adapted the strain in minimal medium containing 0.5% unprocessed corn stover, and 0.025% kraft lignin using three parallel cultures at 60 ˚C and pH 7 under shaking conditions at 150 rpm. After 12 transfers, laccase production significantly improved in the adapted strains of *Geobacillus* sp. strain WSUCF1 (eWSUCF1), from 1.82 ± 0.098 U/mL (the highest obtained with parental unadapted strain in the optimized media) to 7.40 ± 0.026 U/mL (Figure 4). However, little change occurred from the 12th to the 14th transfer (7.40 U–8.88 U), and further transfers had no effect on laccase activity. Furthermore, during the ALE experiments, the evolved strain (eWSUCF1) exhibited significantly improved growth, with maximum laccase production and activity being achieved in 6–7 days, in contrast to 9–10 days for the unadapted strain of WSUCF1 (Figure 2b). We also tested whether the evolved strains still needed kraft lignin as an inducer in the media, and we found that eWSUCF1 very well expressed the laccase enzyme at high concentrations (8.23 ± 0.089 U) with unprocessed corn stover as the substrate, without requiring any extra inducer (data not shown). These outcomes demonstrated that the evolved *Geobacillus* sp. strain WSUCF1 (eWSUCF1) can produce laccase enzyme with higher activity, and in a comparatively shorter time, without requirement for any inducer in the medium, which indicates higher fitness in the adapted population. By the end of 15 cycles of adaptation lasting 6 months, we were able to achieve an 80% enhancement in the enzyme production under batch conditions. This work has practical importance, as this fast adaptation of the *Geobacillus* sp. strain WSUCF1 strain suggests the possibility of growing industrial quantities of *Geobacillus* sp. strain WSUCF1 cells as biocatalysts on reasonably inexpensive carbon sources for commercial use.

### 3.5. Evolution Stability

**Figure 5:**
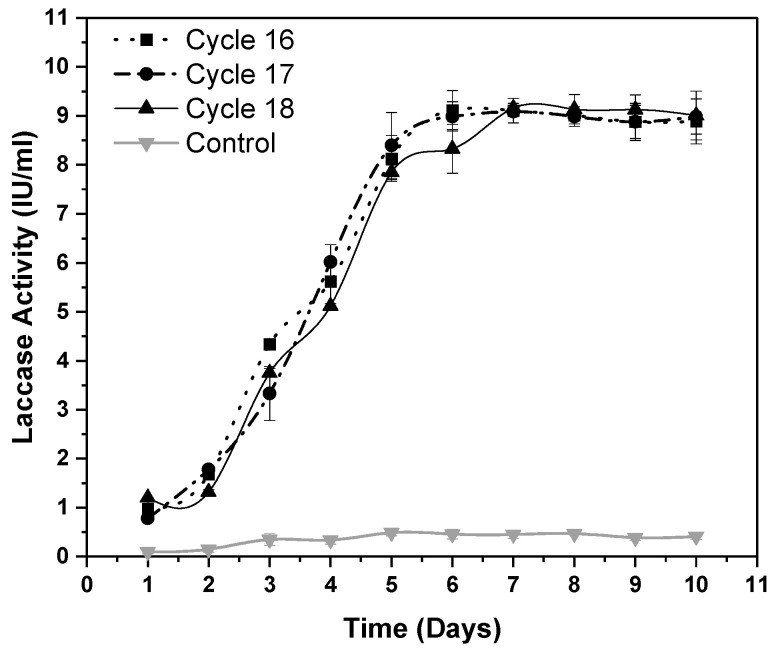
Maximal laccase activity in adapted strains of *Geobacillus* sp. strain WSUCF1 (eWSUCF1) during laboratory adaptive evolution with 0.5% (*w/v*) unprocessed corn stover (CS) and 0.025% (*w/v*) kraft lignin, at 60 °C, pH 7.0, and agitation speed of 150 rpm, after 10 days of incubation. Here, IU = International Units. Note: All the experiments were performed in triplicates, with data averaged and presented as the mean ± standard deviation (SD).

Our first round of ALE experiments was carried out in a period of 6 months, from January to June 2019, and to check for the stability of our evolved population, the experimental set up was again repeated in December of 2019 (after 6 months). The result is shown in Figure 5 where it can be seen that our evolved population of adapted strains of *Geobacillus* sp. strain WSUCF1 (eWSUCF1) was able to retain its stability and activity, with laccase expression reaching 9.12 ± 0.4; 8.99 ± 0.3; and 9.16 ± 0.32 U/mL, during cycles 16,17, and 18, in an expression media containing 0.5% unprocessed CS at pH 7.0, a temperature of 60 °C, and an agitation speed of 150 rpm (Figure 5).

### 3.6. Analysis of Biomass Degradation

To evaluate the lignocellulose degradation efficiency of the adapted *Geobacillus* sp. strain WSUCF1 (eWSUCF1), when compared to the parental strain of WSUCF1, the chemical composition of the corn stover (CS) was analyzed by treating 0.5%(*w/v*) of the substrate with 5% (v/v) inoculum of the respective strain for 10 days at 60 °C, pH 7.0, and agitation speed of 150 rpm. The results of the chemical composition analysis (Table 1) demonstrate that when used as a biocatalyst (at 5% *w/v* inoculum), the adapted strain of *Geobacillus* sp. strain WSUCF1 (eWSUCF1) was able reduce the original lignin and hemicellulose mass fractions, relative to unprocessed corn stover, by 22% and 15% respectively. By comparison, the parental strain of *Geobacillus* sp. strain WSUCF1, before adaptation, was able to remove significantly less of these components, reducing the original lignin and hemicellulose mass fractions by 6% and 9.5%, respectively. Both the adapted and parental strains reduced the cellulose mass fraction by only about 3%.

**Figure 6:**
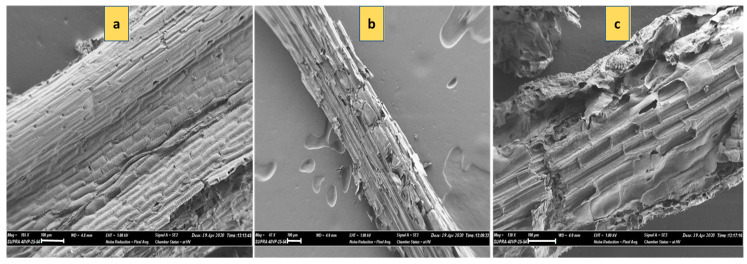
Scanning electron microscopy (SEM) images (scale bar 100µm) of unprocessed corn stover after 10 days of incubation at 60 °C, pH 7.0, and agitation speed of 150 rpm with (**a**) Control with 0% inoculum; (**b**) 5% inoculum of parental strain of WSUCF1; (**c**) 5% inoculum of *Geobacillus* sp. strain eWSUCF1.

Similar levels of microbially-induced degradation have been observed by others; for example, using chemical composition analysis of rice straw, Sheng et al. (2018) demonstrated that lignin removal was up to 22.4%, and cellulose and hemicellulose loss was 13.3% and 17.1%, respectively [26]. In another study, the relative content of lignin decreased from 22.6% to 17% and 19.8% after biotreatment of corn stover with *Trametes hirsute* and *Myrothecium roridum*, respectively [27]. In conventional physicochemical pretreatment processes, abundant cellulose and hemicellulose loss is inevitable. By comparison, during biological pretreatment, the degradation characteristics of lignocellulose are dependent on the different microbes adopted [26]. The degradation results reported here do appear to demonstrate that the adaptive strain of the *Geobacillus* sp. WSUCF1 was more efficient in removing lignin from corn stover, compared to its unadapted version. A qualitative SEM analysis, employed to give some insight into the structural modifications of corn stover after microbial treatment, provides clear evidence of corrosion of the corn stover samples that were biotreated with the WSUCF1 strains (Figure 6b,c) compared to the relatively smooth and flat surface of the untreated corn stover (Figure 6a).

## 4. Discussion

Overall, there are very few examples in the literature where laccase yield obtained from native thermophilic sources are good enough for commercial application. Thus far, the highest extracellular laccase activity reported in the literature from thermophiles includes 55.86 U/mL from *Brevibacillus* sp. Z1 (60 °C) [28], 50.07 U/mL from *Anoxybacillus gonensis* P39 (60 °C) [29], and 11.2 U/mL from *Bacillus* sp. PC-3 (60 °C) [30]. Other reports coming from thermophilic strains indicate a much lower laccase activity; for example, 4.96 U/mL from *Thermobifida fusca* [31], 2.13 U/mL from *Bacillus* sp. PK4 [32], 1.44 U/mL from *Bacillus tequilensis* SN4 [33], 0.076 U/mL from *Streptomyces lavendulae* REN-7 [34], and 0.01 U/mL from *Bacillus* sp. strain WT [35], etc. To sidestep the trouble of low laccase expression by the native thermophilic hosts, various approaches to the heterologous expression of laccases in easily cultivable hosts have been exercised, including the use of multiple gene copies, strong promoters and efficient signal sequences [36]. Systems metabolic engineering is an alternative approach that is proving its worth for the design of novel biocatalysts with improved functionality. In the field of enzyme production, enzymes have been improved by directing their evolution in the laboratory using existing engineering technologies, which involve iterations of random mutagenesis or recombination followed by screening or selection [37], novel expression systems such as cell-surface display [38], modeling of bacterial metabolism [39], and improved fermentation methods [4]. However, unlike rational protein engineering techniques that focus on changing one enzyme property at a time, including directed modification of specific enzymes, ALE has the advantage of letting nonintuitive beneficial mutations occur in many different genes and regulatory regions simultaneously [40]. This accommodates metabolic engineering of microorganisms through a combination of genetic variation and the selection of useful mutations in an impartial fashion, without the requirements of metabolic or regulatory details of the strain. This has a particularly large biotechnological advantage when it comes to engineering enzymes natively in thermophiles.

Thermophiles, in particular the genus of *Geobacillus,* represent non-model organisms with inadequate genetic tools compared to those available for *Escherichia coli, Bacillus subtilis*, or *Saccharomyces cerevisiae* [41]. This is the reason that there is a dearth of studies concerning use of genetic tools to modulate enzyme gene clusters or upstream regulatory factors in thermophiles, despite the increasing profile of these organisms for their catalytic tractability, predominantly in the degradation of second-generation lignocellulosic materials. Since thermophiles are not amendable to metabolic engineering, ALE represents an important alternative strategy for controlling the catabolite flexibility of thermophiles for biotechnological applications, improving yields, and reducing costs in industrial settings. One recent review by Suzuki, 2018, which discusses the peculiarities of environmental adaptations, very strongly indicates the strong capability of *Geobacillus* spp. for environmental adaptation via genome diversification. According to the author, when *Geobacillus* sp. is exposed to an environmental stressor, it differentiates its genomes via stress-induced mutagenesis and transposable elements resulting in the production of derivative cells that are adaptive to the stressor [42]. It is because of this that *Geobacillus* sp. can effectively create variant genes coding for enzyme mutants from the indigenous enzyme genes under suitable selection, and this provides classical evidence of the thermophile’s potential applications in evolutionary-protein engineering.

To date, there has been only one example of the application of ALE to upgrading the performance of a thermophilic microbial strain, in which Zhou et al., 2016, engineered a strain of *G. thermoglucosidasius* to competently produce ethanol from glucose and cellobiose using a combination of metabolic engineering and evolutionary strategies [41]. In the present work, it was demonstrated that, in comparison to use of genomic approaches to mutate a *Geobacillus* for desired features, ALE experiments with these bacteria are easy to establish, especially using batch cultivation in shake flask. Thus, ALE can be performed with the advantages of simplicity of setup, inexpensive equipment, and ease of massive parallel cultures. Furthermore, with ALE in batch culturing, several environmental factors can be easily controlled, including temperature and spatial culture homogeneity (by constant mixing of the culture) [43]. In our work, we used these advantages to optimize the media and a few other critical factors, before we started our serial adaptation approach. This preoptimization of factors before application of ALE in *Geobacillus* sp. strain WSUCF1 may have helped us achieve an approximately 20-fold improvement (0.46 ± 0.04U/mL to 9.23 ± 0.6 U/mL) in the laccase production in just 15 cycles of adaptation, where a typical ALE experiment is performed for somewhere between 100 and 2000 generations [43]. Indeed, work by Suzuki, 2018, provides support for this fast adaptation, mentioning that inductive mutations in *Geobacillus* sp. happen more rapidly than the stress-induced mutagenesis witnessed in other microorganisms [42].

**Figure 7:**
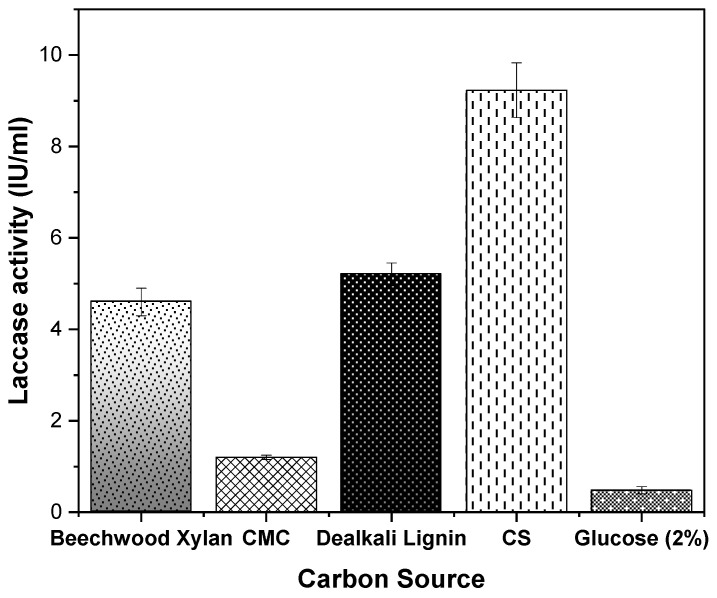
Laccase production in the evolved population of *Geobacillus* sp. strain WSUCF1 (eWSUCF1) at 60 °C, pH 7.0, and agitation speed of 150 rpm, after 10 days of incubation with 0.5% (*w/v*) unprocessed corn stover (CS) as the carbon source, in the presence of different carbon sources as inducers. Here, CMC = Carboxymethyl cellulose, IU = International Units. Note: All the experiments were performed in triplicates, with data averaged and presented as the mean ± standard deviation (SD).

Next, when adaptive evolution takes place, the values of a certain trait change are associated with increased (Darwinian) fitness. Intrinsically, an improved phenotype is often equated to increased fitness of the adapted strain, and this improved fitness of the adapted variant will be apparent through its increased frequency in the total population, during direct competition with an ancestral microbial strain [43]. During ALE, a number of phenotypes occur initially and strive for ‘dominance’ in the total population. Stable phenotypes are meant to accumulate rapidly, but it cannot be assumed that a homogenous population is present during any point of a laboratory evolution experiment. In other conditions of stress or other selective conditions, significant tradeoffs can happen between the adapted and unadapted versions of the strain, and what matters at the end of an ALE experiment is to generate a population that is well evolved to survive and efficaciously reproduce in its environment through the aforementioned selections. To test this adapted population further, we designed different media containing alternative carbon sources without corn stover: beechwood xylan (0.5%); CMC (0.5%); and dealkali lignin (0.05%). Cells were pre-grown on LB agar plates before inoculating into each medium containing a different carbon source, and laccase activity after the 10th day is reported for each medium in Figure 7. While there was not high laccase activity in the presence of CMC (1.2 ± 0.05 U/mL), surprisingly strong laccase activity was observable in the presence of beechwood xylan (4.6 ± 3 U/mL) and dealkali lignin (5.2 ± 0.25 U/mL), although at 50% of the maximum activity observed when the medium contained 0.5% *w/v* CS alone (9.23 ± 0.6 U/mL). Since the adapted strain of *Geobacillus* sp. strain WSUCF1 (eWSUCF1) was producing laccase even in the absence of any extra inducer (kraft lignin) with media containing just corn stover (0.5% as the only carbon source available to the bacteria). Therefore, corn stover (a lignocellulose) has been annotated as a substrate in this manuscript with kraft lignin as the inducer. These results indicate that expression of the laccase enzyme in *Geobacillus* sp. strain WSUCF1 is controlled through the combined effects of soluble nutrients and inducers released from (hemi) cellulose and/or lignin by biodegradation. This means that the higher the amount of lignin and hemicellulose in a lignocellulosic material, the better it is suited for laccase induction in *Geobacillus* sp. strain WSUCF1. This points to a possible alternative explanation for the relatively low laccase induction of pinewood (Figure 1a): It could be that the lower laccase activity induced by PW, relative to CS and PCG, results from the lower *combined* amount of hemicellulose and lignin in PW, compared to cellulose. However, this inference is yet to be confirmed. In future, it would be interesting to decipher the regulatory mechanisms at the genetic and epigenetic level controlling laccase production in this *Geobacillus* strain.

Interestingly, in the medium where glucose is the sole carbon source, a laccase activity of 0.48 ± 0.08 U/mL was recorded. Thus, the expression level of laccase in the adapted strain of *Geobacillus* sp. strain WSUCF1 (eWSUCF1), without the presence of a lignocellulosic material as the inducer, matches the level of laccase that was observed in the ancestral strain of *Geobacillus* sp. strain WSUCF1 when it was first grown in the presence of corn stover (0.46 ± 0.04) U/mL (Figure 1a). This implies that in the case of *Geobacillus* sp. strain WSUCF1, a constitutive level of laccase is always produced, but an enhanced level of enzyme production is observed in the presence of inducers. Such a behavior can point to the presence of two separate genes for laccase in *Geobacillus* sp. strain WSUCF1, with the expression of one gene following the constitutive mechanism, and the second gene being regulated by induction. This leads us to the concept of the occurrence of laccase isozymes, a phenomenon which is commonly observed in many fungal strains [14,44,45], but has not been elucidated so far in bacteria. Our literature search did not find a single report on the presence of laccase isozymes in any bacterium, so our concept of laccase isozyme in *Geobacillus* remains hypothetical and needs to be investigated. Nevertheless, the presence of high laccase activity in the evolved population of *Geobacillus* sp. strain WSUCF1 confirms the success of ALE in adapting the *Geobacillus* sp. strain WSUCF1 to produce and secrete laccase, wherein the evolved changes are quite stable. Our primary aim was to improve the growth profile of *Geobacillus* sp. strain WSUCF1 in the presence of various lignocellulosic substrates, so that the host strain itself can be used as a biocatalyst for consolidated bioprocessing of unprocessed (without pre-treatment) lignocellulosic into various value-added products. Therefore, in this work, the concept of applying ALE was adopted directly for the host strain, *Geobacillus* sp. strain WSUCF1. As follow up work, it would be of interest to apply ALE to the recombinant strains of *E. coli* that were cloned with the laccase gene from *Geobacillus* sp. strain WSUCF1 to express the laccase enzyme.

Overall, our work introduces a non-GMO strategy that has the potential to increase laccase expression in thermophilic bacterial strains. Largely, these findings suggest that adaptive evolution is a viable strategy for the non-recombinant alteration of the existing natural characteristics of a *Geobacillus* sp. ALE simply mimics nature by random mutation of the microorganisms’ own genes, accompanied by selection under suitable conditions to favor the desired phenotype. Recognition is essential, but this is still at the proof-of-concept stage. Further work is required to identify the genetic signatures of these traits.

## 5. Future Work

In follow up work to this research, we are currently performing multiple omics (transcriptomics, proteomics, and epigenomics) measurements to investigate systematically the genetic and phenotypic changes in the evolved trains of *Geobacillus* sp. strain WSUCF1 (eWSUCF1). Additionally, we plan to perform next-generation sequencing studies of the wild-type as well as its evolved eWSUCF1version, to identify all the mutations and understand how the various mutations combine to form an adaptive trait, and the results from these analyses will be a part of our future manuscript on the subject. Together, this comprehensive data will provide valuable insights into the enzyme systems of a lignocellulolytic strain *Geobacillus* sp. strain WSUCF1 during growth on corn stover and will also reveal the dynamics of enzyme complements during the lignocellulose deconstruction process.

## 6. Conclusions

*Geobacillus* sp. strain WSUCF1 can rapidly and effectively hydrolyze unprocessed lignocellulosic biomasses, and this makes this strain a promising candidate for lignocellulolytic enzymes in view of the demand for thermostable enzymes in the biotechnology industry. In the present work, we employed an evolutionary approach to improve growth and laccase expression in *Geobacillus* sp. strain WSUCF1 by serial adaptation in minimal media containing untreated corn stover as the substrate, and kraft lignin as the co-substrate. This minimal media was specifically optimized for enhanced laccase production in this native strain, after testing various agricultural waste residues. After 15 transfers, laccase production significantly increased in the evolved *Geobacillus* sp. strain WSUCF1 (eWSUCF1), from 0.46 ± 0.04 U/mL (the highest obtained with the parental, unadapted strain) to 9.23 ± 0.6 U/mL. In future, it would be interesting to decipher the regulatory mechanisms at the genetic and epigenetic level controlling laccase production in this strain of *Geobacillus* sp.

## Figures and Tables

**Table 1:** Chemical analysis of the corn stover composition.

Component	Initial CS Contents, Dry Basis (wt. %)	CS Contents after Treatment with Parental WSUCF1, Dry Basis (wt.%)	Mass Change after Treatment with Parental WSUCF1, Dry Basis (%)	CS Contents after Treatment with eWSUCF1, Dry Basis (wt. %)	Mass Change after Treatment with eWSUCF1, Dry Basis (%)
Lignin	18.5	17.4	−5.9	14.4	−22.2
Hemi-Cellulose	33.6	30.4	−9.5	28.6	−14.9
Cellulose	29.4	28.6	−2.7	28.4	−3.1
Extractives and solubles	18.4	22.6	+22.8	27	+46.7
Ash	0.1	1.02	+9.2	1.56	+14.6

Here, CS = corn stover; parental WSUCF1 represents unadapted strain of *Geobacillus* sp. strain WSUCF1; eWSUCF1 represents adaptive *Geobacillus* sp. strain WSUCF1.
